# Enhancing the Performance of Evolutionary Algorithm by Differential Evolution for Optimizing Distillation Sequence

**DOI:** 10.3390/molecules27123802

**Published:** 2022-06-13

**Authors:** Zehua Hu, Peilong Li, Yefei Liu

**Affiliations:** 1 College of Chemical Engineering, Nanjing Tech University, Nanjing 211816, China; merlinihu0828@gmail.com; 2 Institute of Materials, China Academy of Engineering Physics, Mianyang 621907, China; lipeilong2012@126.com

**Keywords:** distillation process synthesis, distillation sequence, evolutionary algorithm, Aspen Plus

## Abstract

Optimal synthesis of distillation sequence is a complex problem in chemical processes engineering, which involves process structure optimization and operation parameters optimization. The study of the synthesis of distillation sequence is a crucial step toward improving the efficiency of chemical processes and reducing greenhouse gas emissions. This work introduced the concept of binary tree to encode the distillation sequence. The performance of the six evolutionary algorithms was evaluated by solving a 14-component distillation sequence synthesis problem. The best algorithm was used to optimize the operation parameters of a triple-column distillation process. The total annual cost and CO_2_ emissions were considered as the metrics to evaluate the performance of triple-column distillation processes. As a result, NSGA-II-DE was found to be the best one of the six tested evolutionary algorithms. Then, NSGA-II-DE was applied to the distillation sequence optimization to find the best operating parameters, which led to a significant reduction in CO_2_ emission and total annual costs.

## 1. Introduction

Design and optimization of distillation sequences become much more essential tasks in process systems engineering, which dramatically influences total annual cost (TAC) and target product purity. The distillation process synthesis was pioneered by Lockhart [1] and systematically analyzed by Siirola et al. [2]. Until now, there have been some popular methods that deal with distillation process synthesis, including the heuristic method, evolutionary method, algorithmic method, and stochastic algorithms. In recent years, many researchers [3,4] focused their attention on distillation process to reduce energy consumption in distillation separation or improve production efficiency, for instance, the coupling of distillation and vapor permeation [5,6] or reactive-vapor permeation-distillation [7]. Although these distillation processes are beneficial to production, design and optimization of distillation sequences are still essential.

Heaven [8] firstly proposed the heuristic method for distillation column optimization. Thompson and King [9] realized automatic calculation of the heuristic method by coding distillation sequences into digits. Freshwater and Henry [10] supplemented inference rules based on Heaven’s method. Seader and Westerberg [11] proposed six inference rules to generate the initial separation sequence. The evolutionary method evolved from the heuristic method, which tries out the optimal structure by changing the separation order of key components. Although the heuristic and evolutionary methods have been widely applied in engineering practice, their effectiveness is critically dependent on the completeness of the design engineer’s knowledge, and there is no guarantee that the design of a distillation sequence will be optimal. The algorithmic methods introduce mathematical models for nonlinear programming to deal with the problem of distillation sequences. Nonlinear programming models obtain globally optimal solutions by the gradient descent method. However, due to their non-convex nature, which consistently exhibits in distillation sequence synthesis, the solution often converges to local extrema.

The stochastic algorithms have an attractive advantage over heuristic and evolutionary methods for problems with the modular simulator, where the model for each unit is only in an implicit form (black box model). Firstly, they are based on direct searching methods so that there is no need to have a rigorous model of the problem, which makes it suitable for the processes which are difficult to be modeled. Secondly, the search for the optimal solution is not limited to a single point instead of relying on multiple points contemporaneously, which makes it particularly suitable for handling combinatorial optimization problems with high computational complexity [12].

In recent years, stochastic algorithms have been widely applied for the synchronous optimization of one or more different types of variables. The prevailing stochastic algorithms for the analysis, design, and evaluation of distillation processes have been used directly in the modeling, thus avoiding the sophisticated modeling and handling mixed-integer non-linear programming (MINLP) [13]. Stochastic algorithms can be classified into two main categories according to the dimensionality of the objective function. In the field of single-objective optimization, Floquet et al. [14] applied the simulated annealing (SA) algorithm with a separation-based coding procedure for complex column sequence synthesis. Leboreiro and Acevedo [15] presented a framework for the synthesis and design of complex distillation sequences based on a modified genetic algorithm (GA) coupled with a sequential process simulator. In the last decade, many researchers have evaluated the distillation sequence in multi-dimension to assess the sustainability of the chemical process. Instead, a multi-objective optimization algorithm has been applied. For the multi-objective optimization, Errico et al. [16] proposed an efficient method for the design and optimization of intensified distillation systems by combining the sequential design method and the multi-objective differential evolution. Vázquez-Castillo et al. [17] presented a multi-objective optimization approach that integrates the design and control of multicomponent distillation sequences. Contreras-Zarazúa et al. [18] studied multi-objective optimization involving costs and control properties of intensified schemes to produce diphenyl carbonate. Cabrera-Ruiz et al. [19] introduced a new strategy to consider controllability as an optimization criterion along with the optimal design at a steady state. Alcocer-García et al. [20] used differential evolution with the tabu list in optimizing TAC and the eco-indicator 99. Sun et al. [21] applied an improved multi-objective genetic algorithm to optimize of the triple-column extractive distillation (TCED) process. Zhang et al. [22] suggested a method to solve multi-objective optimization (MOO) in the Fischer–Tropsch reactive distillation synthesis. Zhao et al. [23] combined sequential iterative optimization sequence and a MOO algorithm to optimize equipment costs and CO_2_ emissions. 

One representative of stochastic algorithms is the so-called evolutionary algorithm, which is widely used for highly non-linear problems. Evolutionary algorithms are a type of stochastic search algorithms which simulate the natural selection and natural evolution of organisms. They mainly include non-dominated sorting genetic algorithm II (NSGA-II) [24], non-dominated sorting genetic algorithm III (NSGA-III) [25], non-dominated sorting genetic algorithm II and III with differential evolution (NSGA-II-DE and NSGA-III-DE) [26], multi-objective evolutionary algorithm based on decomposition (MOEA/D) [27], and multi-objective evolutionary algorithm based on decomposition and differential evolution (MOEA/D-DE) [28]. Although a few studies have been carried out for distillation process synthesis with evolutionary algorithms, nobody has clarified the performances of different evolutionary algorithms, resulting in a lack of guidance for evolutionary algorithms. Furthermore, the role of differential evolution has never been discussed in distillation process synthesis.

In the present study, six evolutionary algorithms were explained and implemented with in-house codes. The performance of the evolutionary algorithms was firstly tested with a 14-component distillation sequence to discern the best one. The effectiveness of NSGA-II-DE was then examined by optimizing the distillation sequence of a triple-column distillation sequence proposed by Mayevskiy et al. [29]. Finally, some opinions on applying this method to general process synthesis and design issues are provided.

## 2. Evolutionary Algorithm

### 2.1. NSGA-II

**Figure 1:**
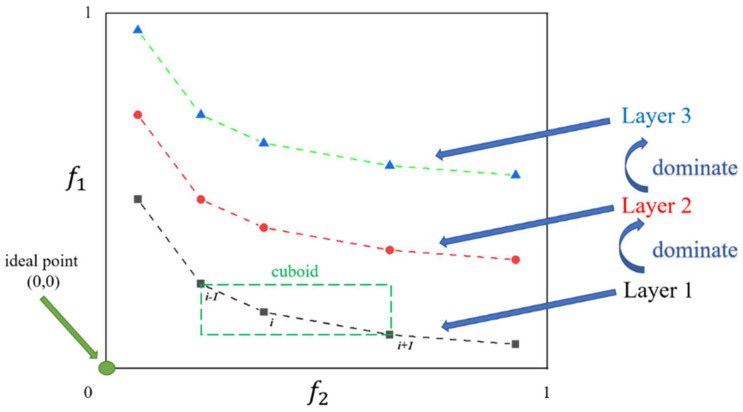
Crowding-distance calculation.

Deb et al. [24] proposed NSGA-II on the basis of NSGA [30]. In NSGA-II, the evolutionary population is divided into several layers according to the dominance relationship. The dominance of the layers is shown in the schematic diagram of crowding-distance calculation (Figure 1). To obtain an estimate of the density of solutions surrounding a particular solution in the population, NSGA-II calculates the average distance of two points on either side of this point along each of the objectives. In Figure 1, the crowding distance of the *i*_th_ solution in its front (marked with black squares) is the average side length of the cuboid (shown with a green dashed box). When new populations are generated, the best individuals with relatively low densities are usually retained and participate in the evolution of the next generation. 

**Figure 2:**
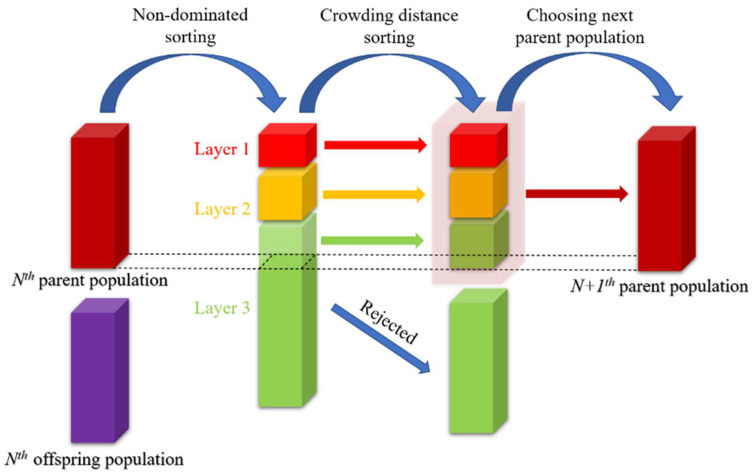
Procedure of NSGA-II.

The whole NSGA-II procedure is illustrated in Figure 2. In the *N^st^* generation of the parent population, individuals in the population are divided into subpopulations that do not interbreed based on non-dominance sorting, and the individuals satisfying a certain number of subpopulations are selected to enter the next generation of the parent population based on their dominance relationships.

### 2.2. NSGA-III

**Figure 3:**
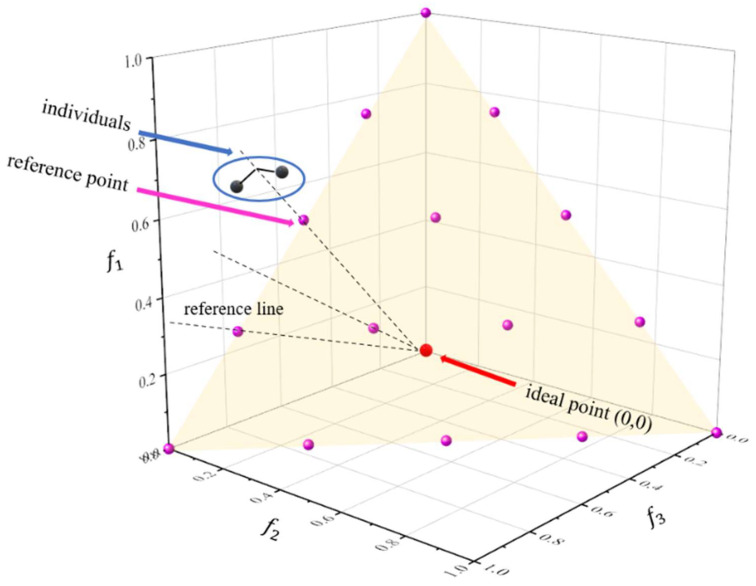
Association of individuals with reference points.

NSGA-II handles multi-objective optimization problems very well. However, it is only available for low-dimensional optimization problems (objective dimension less than or equal to 3). As the objective dimension of the optimization problem increases, the number of non-dominated individuals in the population grows exponentially, which makes it challenging to distinguish good and bad individuals in the Pareto dominance. Deb and Jain [25] proposed NSGA-III to solve this problem. The framework of NSGA-III is similar to that of NSGA-II, except that NSGA-III introduces reference lines for the non-dominance ranking of individuals, as shown in Figure 3. 

### 2.3. MOEA/D

**Figure 4:**
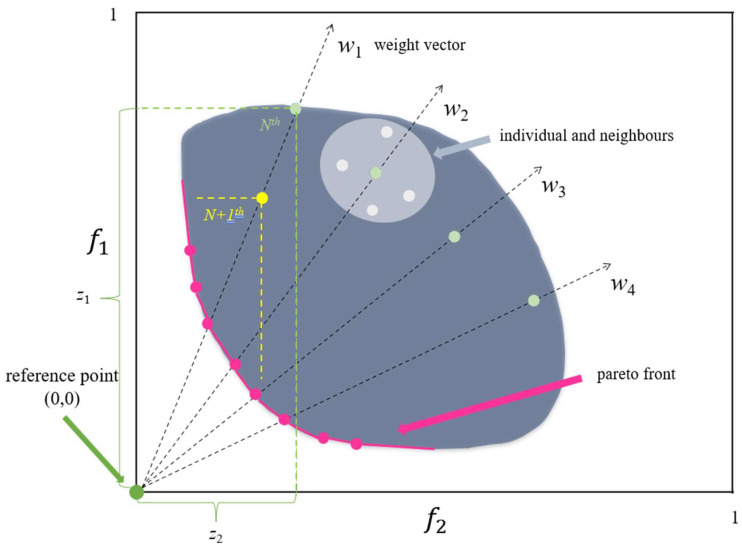
The procedure of MOEA/D.

The core principle of the MOEA/D algorithm is to decompose a multi-objective optimization problem into a set of single-objective sub-problems or multiple multi-objective sub-problems, which finds an approximation to the entire Pareto surface (pink line in Figure 4) by using the neighbor relationships between the sub-problems to optimize all the sub-problems simultaneously in a collaborative manner [27]. Typically, the sub-problems are defined by weight vectors (black dashes in Figure 4), and the neighbor among sub-problems is determined by calculating the Euclidean distance between the weight vectors. Sum functions which are commonly applied are the weighted sum approach [31], Tchebycheff approach [32], and the penalty-based boundary intersection approach [27]. The MOEA/D algorithm used in this work is based on the implementation of the Tchebycheff approach. The result of the Tchebycheff sum function *g^tch^* is calculated as:

(1) $$g^{tch}(x|w)=\underset{i\in \{1,\ldots ,m\}}{\max}w_{i}\left|f_{i}(x)-z_{i}\right|$$

where *i* is individual; *z_i_* is the value of the reference point *z*, *x* is the decision vector, *w* is the weight vector.

### 2.4. Differential Evolution with NSGA-II, NSGA-III, and MOEA/D

The differential evolution (DE) algorithm is based on population differences, which finds solutions to optimization problems through cooperation and competition between individuals [33,34]. The concept of differential evolution is as follows:

Firstly, two individuals (*x_r1_*, *x_r2_*) are randomly selected from the population, and the difference between the individuals is multiplied by the differential weight (*F*), and they are added to the third individual (*x_r3_*) to generate a mutant individual (*v_i_*):

(2) $$v_{i}=x_{r3}+F\left(x_{r1}-x_{r2}\right)$$

where *v_i_* is mutant individual, *x_r1_*, *x_r2_,* and *x_r3_* are selected individuals, *F* is differential weight.

Secondly, the mutant individual will replace the original individual in the population with a certain probability so that a test population is created:

(3) $$u_{i}=\{\begin{array}{cc} v_{i} & \;\;\mathrm{random}(0,1)\leq Cr\;\;\mathrm{or}\;\;i=i_{random}\\ x_{i} & \mathrm{random}(0,1)>Cr \end{array}\;$$

where *u_i_* is text individual, *v_i_* is mutant individual, *x_i_* is original individual, *Cr* is crossover factor. Finally, the test population is compared with the original population and better ones are retained into the next generation.

It has been found that differential evolution strategies, when introduced into multi-objective optimization algorithms, will improve the global search capability and convergence of multi-objective optimization algorithms [35,36]. However, no one has introduced a genetic algorithm based on differential evolution to the distillation sequence synthesis problem.

## 3. Binary Tree Coding

For ease of representation and calculation, the following two assumptions were introduced into the design of distillation sequences: (1) All components in the flow unit were arranged in ascending order according to their boiling points. (2) Each unit consists of a simple tower, and the splitting of the material is clearly split, i.e., the distillation column had only one inlet flow unit and two outlet flow units at the top and bottom of the tower, and both of them were capable of separating the light and heavy key components.

**Figure 5:**
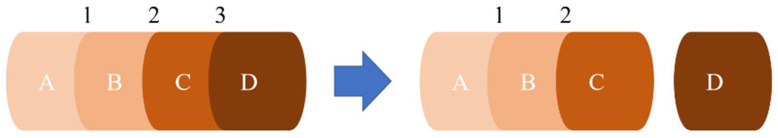
Separation number of four components.

For a mixture of four components A, B, C, and D, the distillation separation sequence can be expressed in the form of ABC|D, with “|” representing the separation task, i.e., the separation of ABCD into two mixtures ABC and D, as shown in Figure 5.

**Figure 6:**
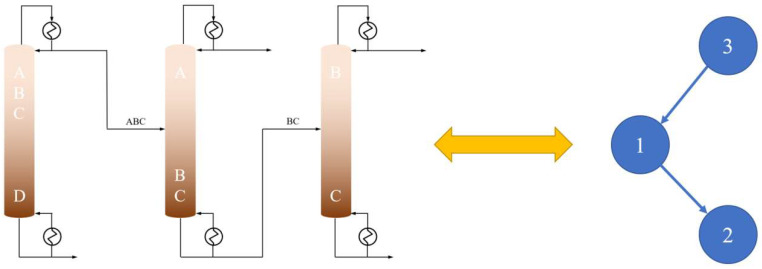
Diagram of separation sequence and binary tree. A, B, C, D are the four components of the mixture and 1, 2, 3 represent the numbers of separation positions.

In order to make the distillation sequence correspond to the array of separation tasks, the distillation sequence was encoded in a binary tree, taking into account the similarity between the structure of a sharp distillation sequence and a binary tree. For example, S [3, 1, 2] is a random task array for a distillation sequence. The three data were ordered into the binary tree, as shown in Figure 6. The child nodes were found through the pre-order traversal, and the parent nodes were found through the post-order traversal. As a result, the number of the downstream distillation columns and upstream distillation columns are determined, respectively. The ordered binary tree was S [3, 1, 2] for the pre-traversal and S [2, 1, 3] for the post-traversal.

## 4. Comparative Study of Evolutionary Algorithms

Due to sufficient thermodynamic data for saturated alkanes, this section illustrates the distillation separation of a 14-component alkane mixture, where a separation sequence needs to be designed to separate the components thoroughly. There are 742,900 possible combinations in a 14-component distillation sequence problem, which is complex enough to test the performance of six evolutionary algorithms. The component numbers, ingredients, molar feeds, boiling points, and K-values are listed in Table 1.

The evaluation metrics of the independent separation units are expressed in terms of annual cost correlations. The optimal synthesis using rigorous simulation analysis is so complex that an evaluation function is usually specified to evaluate the designed sequence for simplicity in the design of the distillation sequence [37]. The evaluation functions are usually relative cost function [38], separation ease coefficient [39], and separation difficulty coefficient [40]. Shi and Wang [38] compared the separation coefficient and the relative cost function, concluding that the relative cost function method is more reasonable as an evaluation index. In order to test the performance of the evolutionary algorithms, relative cost function and separation difficulty coefficient were adopted as the objective functions in this work.

The relative cost function (*F*) is expressed as

(4) $$F=\sum_{i}F_{i},F_{i}=\left\{(1-func{)}^{2.73}+2.41\right\}\Delta T_{b}^{-0.31},func=\left\{ \begin{array}{ll} t/w & t\leq w\\ w/t & t>w \end{array}\right.$$

where Δ*T_b_* is the boiling point difference between the light and heavy key components, *t* is the distillate rate, *w* is the bottom rate.

The separation difficulty coefficient (*C_DS_*) is expressed as

(5) $$C_{DS}=\frac{\mathrm{lg}\left[{\left(\frac{x_{lk}}{x_{hk}}\right)}_{D}/{\left(\frac{x_{lk}}{x_{hk}}\right)}_{W}\right]}{\mathrm{lg}\alpha_{lk,hk}}\cdot \frac{t}{t+w}\cdot \left\{1+\left|\frac{t-w}{t+w}\right|\right\}$$

where ${\left(\frac{x_{lk}}{x_{hk}}\right)}_{D}$ is the mole fraction ratio of light and heavy key components in distillate product, ${\left(\frac{x_{lk}}{x_{hk}}\right)}_{W}$ is the mole fraction ratio of light and heavy key components in bottom product, $\alpha_{lk,hk}$ is the relative volatility of light and heavy key components, *t* is the distillate rate, kmol/h, *w* is the bottom rate, kmol/h.

The parameters used in the evolutionary algorithms are listed in Table 2. The optimization procedure was carried out on a 64-bit desktop computer with an Intel Core i7-12700 CPU @2.10 GHz, including a 16 GB RAM. Considering that multi-objective optimization algorithms are characterized by random evolution, the performance of algorithms may not be indicated by the results of a single calculation. Therefore, the calculation was repeated 100 times in this paper, and the results were statistically analyzed to find out the pattern and characteristics.

Where *dim* is dimension of the objective function, *size* is size of population, *gen* is generation, *cf* is crossover factor, *mf* is mutation factor, *ps* is probability of selection, *sn* is size of neighbor, *f* is scaling factor in differential evolution operator.

The evaluation of the multi-objective evolutionary algorithm (MOEA) takes into account two main indicators: effectiveness and efficiency. The effectiveness refers to the quality of the Pareto optimal solution set it finds, mainly in terms of the convergence and distribution effect of MOEA. The efficiency refers to the CPU time it takes to find the Pareto solution set for a multi-objective optimization problem, and the RAM it occupies. The comprehensive evaluation metric reflects both convergence and distribution of MOEA through a scalar value. In recent years, hypervolume (HV) [41] and indicator generational distance (IGD) [42] have been widely used, and both of them are applied in evaluating the effectiveness of evolutionary algorithms.

**Figure 7:**
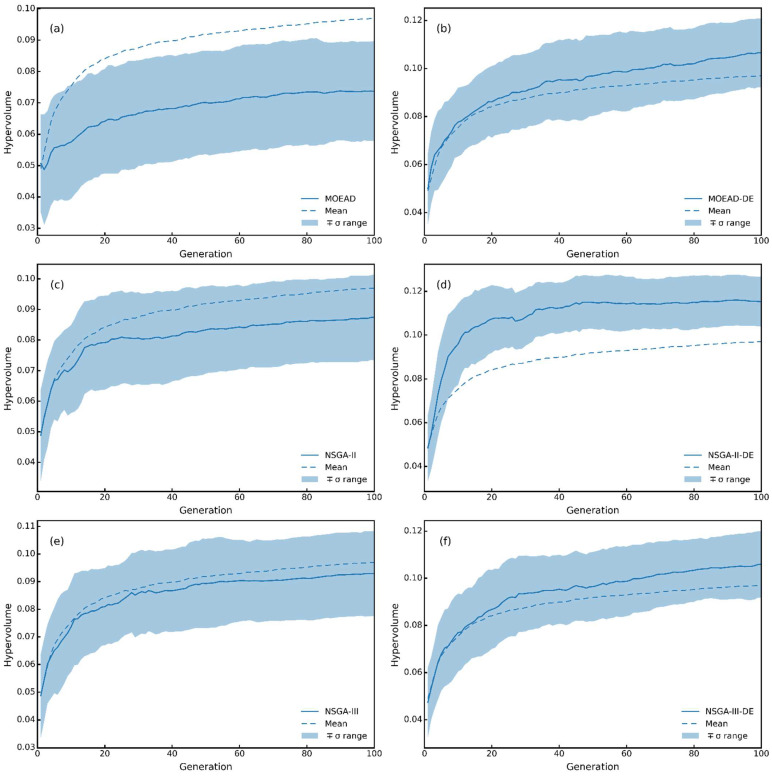
Hypervolume. (**a**) MOEAD; (**b**) MOEAD-DE; (**c**) NSGA-II; (**d**) NSGA-II-DE; (**e**) NSGA-III; (**f**) NSGA-III-DE.

Figure 7 shows the change in HV with the evolution of the population. The Blue dashed line represents the mean HV values of six MOEAs in each generation. The blue-filled part indicates the fluctuation of the result, which was calculated from *μ* ± *σ*, where *μ* is mean HV and *σ* is standard deviation. The HV value was strictly subject to the Pareto dominant principle. For example, if individual A dominates individual B, the HV value of individual A must be greater than that of individual B. As the differential evolution strategy enhanced the search ability of evolutionary algorithm, the performance of the three evolutionary algorithms was improved. At the same time, the HV value of NSGA-II-DE was significantly higher than the others, which means NSGA-II-DE is optimal in terms of the analysis results of the HV indicator.

**Figure 8:**
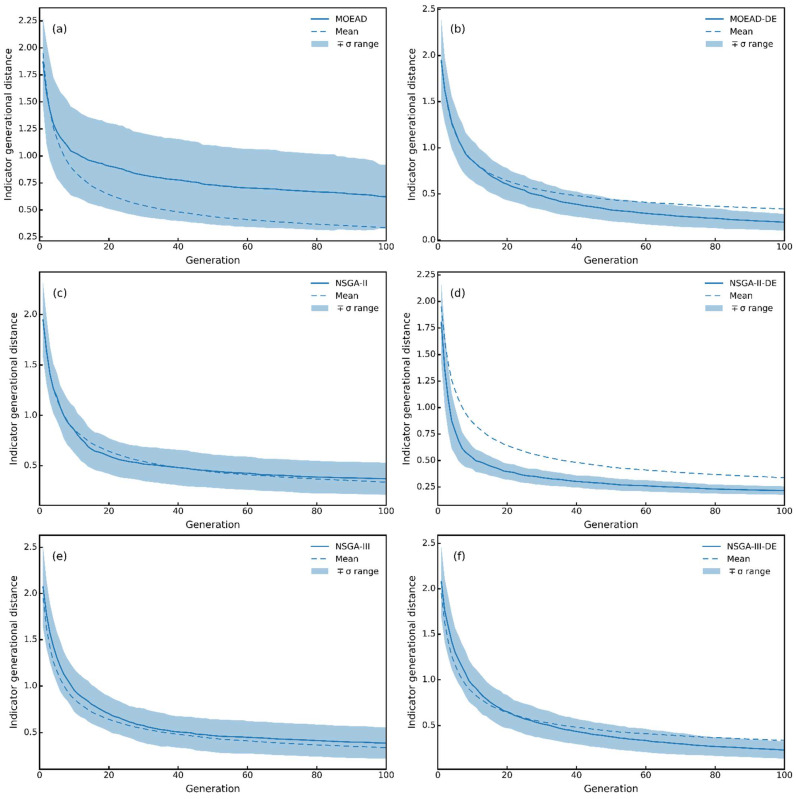
Indicator generational distance. (**a**) MOEAD; (**b**) MOEAD-DE; (**c**) NSGA-II; (**d**) NSGA-II-DE; (**e**) NSGA-III; (**f**) NSGA-III-DE.

Figure 8 shows the change in IGD with the evolution of the population. The IGD value indicates the average distance from the individuals in the Pareto set to the non-dominated solution set found by the evolutionary algorithm, which means that the smaller the value of IGD, the better the performance of the algorithm. Since the IGD value is related to the preset Pareto front curve and the Pareto front curve cannot be directly derived, an approximation was used instead of the Pareto front curve, which may have affected the IGD values of the six algorithms. Thus, the performance of the four algorithms, except for the NSGA-II –DE and MEOA/D, was considered to be comparable.

**Figure 9:**
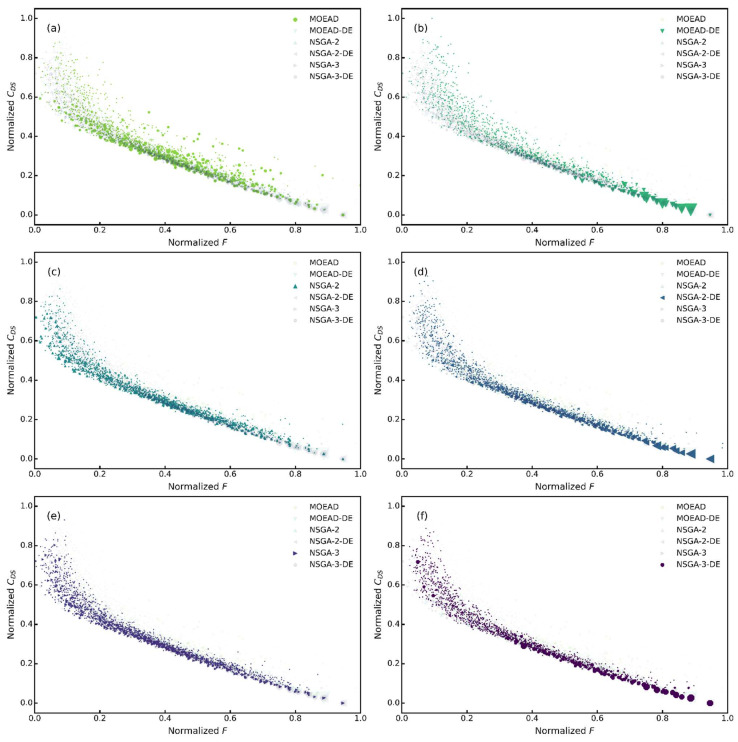
Non-dominated fronts. (**a**) MOEAD; (**b**) MOEAD-DE; (**c**) NSGA-II; (**d**) NSGA-II-DE; (**e**) NSGA-III; (**f**) NSGA-III-DE.

From Figure 9, the size of the marker indicates the probability of recurrence 100 times. If more individuals converge to the same point, the larger the marker will be, which means a higher performance level of the algorithm. It can be seen that the non-dominated front calculated by NSGA-II-DE outperformed the non-dominated front obtained by the other algorithms, which led to the conclusion that the NSGA-II-DE algorithm performs better than the other algorithms. The convergence and distribution of NSGA-II, NSGA-III, and MOEA/D were improved by introducing the differential evolution operator as an evolutionary strategy, which is consistent with the findings reported in the literature [35,36].

## 5. Optimization of the Base Case

### 5.1. Separation Flowsheet and Thermodynamic Modeling

This present study introduced a separation scheme proposed by Mayevskiy et al. [29] instead of redesigning a distillation sequence to separate the 6-composition mixture. The same as the reference publication, a feed flow of 100 kmol/h with molar compositions of 0.4934, 0.1717, 0.0847, 0.1927, 0.0015, and 0.0575 for acetone, isopropanol, water, methyl isobutyl ketone (MIBK), methyl isobutyl carbinol(MIBC), and diisobutyl ketone(DIBK), respectively was used.

**Figure 10:**
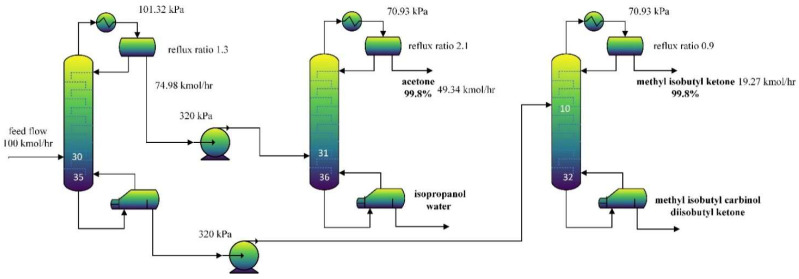
Flowsheet of 6-component mixture separation.

The first column plays a role in pretreatment. The high-boiling-point components (MIBK, MIBC, DIBK) and low-boiling-point components (acetone, isopropanol, water) were divided from this column. The products of distillate and bottoms were refined in the second and third columns. A pump conveyed the stream, and the pump discharge pressure was set at 320 kPa. The last two distillation columns were operated under negative pressure. However, because the ratio *C_P_*/*C_V_* for MIBK, MIBC, and DIBK (1.048, 1.045, 1.033) is relatively small, this may lead to higher operating costs for the vacuum pump. Thus, the operative pressure of the distillation column will be redesigned. The distillation sequence to be optimized is shown in Figure 10. More details can be found in Mayevskiy et al. [29].

The above process was simulated by Aspen Plus v11. Design specifications were set in the RadFrac block to ensure that the product purity met the separation requirements. In the pretreatment column, molar recoveries of isopropanol in distillate and MIBK in the bottom were both 0.9995. The others were set molar purity of key components as design specifications.

To be consistent with the simulation approach in the publication, Non-Random Two-Liquid (NRTL) was chosen as the thermodynamic model. The validity of the NRTL model has been discussed in the literature [29]. However, the single activity coefficient model is not able to calculate the thermodynamic state of the gas. When it occurs, the Aspen Plus simulator uses ideal gas law to calculate the gas thermodynamic state, which compensates for this deficiency, but it can introduce theoretical errors. NRTL parameters for binary systems are shown in Table 3.

### 5.2. Performance Indicators

In order to evaluate the performance of the distillation sequence, total annual cost and CO_2_ emissions were adopted as indicators in this work [43,44]. The total annual cost is a commonly used economic indicator which includes capital investments and operating costs. In the present work, the capital investments consisted of column vessel cost, column tray cost, vacuum pump cost, heat exchanger cost, and feed pump cost. Electricity consumption, cooling water consumption, and steam consumption influence operating costs.

The total annual cost is calculated as

(6) $$\mathrm{TAC}=\frac{\mathrm{capitalinvestments}}{\mathrm{payback}\;\mathrm{period}}+\mathrm{operating}\;\mathrm{cost}$$

where the payback period is 3 years.

The column cost is calculated as

(7) $$C_{c}=\frac{MS}{280}\times 101.9\times D_{c}^{1.066}\times L_{c}^{0.802}\times \left(2.18+3.67\right)$$

where *D*_c_ is the diameter of the column (ft); *L_c_* is the height of the column; *MS* is the Marshall & Swift index. Here, M&S was taken as 1448.3. The height of the column is calculated as

(8) $$L_{c}=2.3\times (N_{T}-1)$$

where *L_C_,* is height of column, [ft]; *N_T_* is the total number of trays in the column.

The tray cost is calculated as

(9) $$C_{T}=\frac{MS}{280}\times 4.7\times D_{c}^{1.55}\times L_{c}^{}\times \left(1+1.8+1.7\right)$$

The cost of heat exchanger is calculated as

(10) $$C_{he}=\frac{MS}{280}\times 5109.49\times A^{0.65}\times \left(2.29+F_{c}\right)$$

where *A* is the area of heat exchanger (ft^2^); *F_C_* is 5.0625 for the reboiler and 3.75 for the condenser. For the reboiler and condenser,

(11) $$A_{R}=\frac{Q_{R}}{U_{R}\Delta T_{R}}$$

(12) $$A_{C}=\frac{Q_{C}}{U_{C}\Delta T_{C}}$$

where *A_R_* and *A_C_* is heat transport area of reboiler and condenser, respectively (m^2^); *U_R_* is the transfer coefficient for re boiler (kW/(K·m^2^)), Δ*T_R_* is the temperature difference, (K). *Uc* is the transfer coefficient for condenser (kW/(K·m^2^)), Δ*Tc* is the temperature difference (K). Here, *U_R_* was taken as 0.568 and *Uc* was taken as 0.852.

The cost of vacuum pump and feed pump are given as [45]

(13) $$C_{vp}=4200\times {\left(\frac{60\times F_{v}\times 8.314\times 273.15}{3600\times 101325}\right)}^{0.55}$$

where *F_V_* is the volumetric flow rate (kmol/h).

(14) $$C_{fp}=26700\times {\left(\frac{24\times F_{F}\times 3600}{50000}\right)}^{0.53}$$

where *F_F_* is the feed flow rate (m^3^/s).

The utility cost is calculated as

(15) $$C_{LP}=7.07\times Q_{LP}\times 8000$$

(16) $$C_{MP}=8.57\times Q_{MP}\times 8000$$

(17) $$C_{CW}=0.354\times Q_{CW}\times 8000$$

(18) $$C_{E}=0.0775\times Q_{E}\times 8000$$

where *C_LP_* is the cost of lower pressure steam (3 bar); *Q_LP_* is the lower pressure steam duty (GJ); *C_MP_* is the cost of medium pressure steam (15 bar); *Q_MP_* is the medium pressure steam duty (GJ); *C_CW_* is the cost of cooling water (298.15 K); *Q_CW_* is the cooling water duty (GJ); *C_E_* is the cost of electricity; *Q_E_* is the electricity duty (kW).

In considering the impact of distillation separation on the environment, this work used Greenhouse gas emissions as an evaluation index. This methodology was proposed by Gadalla et al. [46] and CO_2_ emissions can be calculated with the following equation.

(19) $${\mathrm{CO}}_{2}\;\mathrm{emissions}=\left(\frac{Q_{Fuel}}{NHV}\right)\left(\frac{C\%}{100}\right)a\;$$

where *Q_Fuel_* is amount of fuel burnt (kW); *a* is the ratio of molar masses of CO_2_ and C; *NHV* represents the net heating value of fuel with a carbon content of C% (kJ/kg).

### 5.3. Optimization Methodology and Objective Function

**Figure 11:**
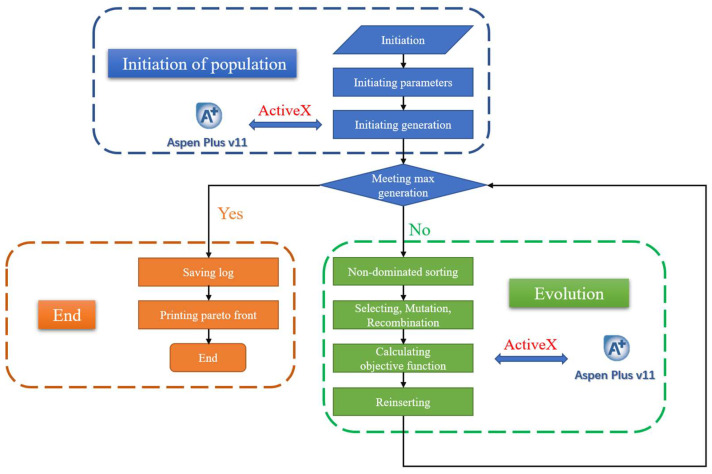
Aspen Plus calculations with NSGA-II-DE for the distillation sequence.

The improved non-dominated sorting genetic algorithm II based on differential evolution algorithm (NSGA-II-DE) was applied to optimize the distillation sequence to provide quantitative benefits and trade-offs between annual operating costs and capital investments. The NSGA-II-DE procedure includes initiation of population, evolution, and end, as shown in Figure 11. The optimization of the distillation sequence is based on the Aspen Plus v11, which enables communication between python and Aspen Plus through ActiveX [47]. The design parameters were generated by a Python program and entered into Aspen Plus simulator via the COM technique. After the simulation was completed without errors, the Aspen Plus simulator returned the results (TAC and CO_2_ emissions) to Python. TAC and CO_2_ emissions were calculated using the calculator module of Aspen Plus. The NSGA-II-DE was used in Python to analyze the corresponding objective functions under different design parameters, evolving until the number of generations satisfied 100 generations.

The two targets mentioned already were accounted for in the objective function, which is defined as follow:

(20) $$\min(\mathrm{TAC},{\mathrm{CO}}_{2})=f(N_{T},N_{F},R,F_{d},P)$$

where TAC is total annual cost (million$); CO_2_ is CO_2_ emissions (kt/year); *N_t_* is the total number of trays in the column; *N_F_* is feed stage, *R* is mole reflux ratio, *F_d_* is distillate rate (kmol/h); *P* is operative pressure (kPa). The objective function is restricted by fulfilling the molar purity of the key component upper than 0.998 or molar recovery of the key component upper than 0.999.

In this example, the feed plate was changed to the ratio of the feed plate to the number of plates, and the result was rounded down to ensure that the feed plate is always smaller than the total number of plates. The total number of design variables, in this case, was 15. To ensure that the molar purity of the product reached 99.8%, the design specifications were set in the RadFrac block. At the same time, the molar recoveries of the key components are charged at 99.9%. There are still nine design variables, which can be found in Table 4.

### 5.4. Optimization Results

**Figure 12:**
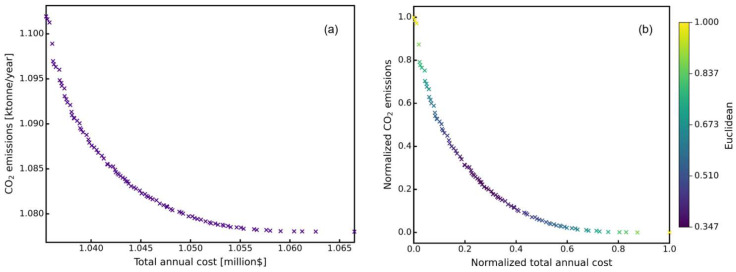
Pareto sets of TAC and CO_2_ emissions: (**a**) raw result and (**b**) normalized result.

In this work, the optimization procedure was carried out on a 64-bit desktop computer with an Intel Core i7-12700 CPU @2.10 GHz, including 16 GB RAM. The parameters of NSGA-II-DE are the same as the ones listed in Table 2. The program took about 8 h to obtain the optimization results. The non-dominated front is shown in Figure 12.

By increasing the number of plates, the separation performance of the distillation column can be improved, and therefore the equipment investment also increases. When the number of plates was increased or decreased to a certain level, there was no significant reduction in CO_2_ emission or total annual cost. At the same time, the total annual cost and CO_2_ emissions were normalized, which makes it possible to directly find the Euclidean distance from the ideal point (0,0) for the results to compare the superiority of the individuals on the non-dominated front. In Figure 12b, the darker the scatter color, the closer to the ideal point.

The optimization results are shown in Table 5. By optimizing the operational parameters of the distillation sequence through NSGA-II-DE, the total annual cost was reduced by 1.868 million$, and CO_2_ emission was reduced by 5.002 kt/year. The CO_2_ emission and total annual cost were reduced by 64.15% and 82.23%. As expected, a reduced-pressure distillation operation in the T3 column would incur significant energy costs, making it more suitable for atmospheric pressure separation.

## 6. Conclusions

This work presented the performance test of six evolutionary algorithms used in previous work. Firstly, the 14-component distillation sequence problem was successfully solved. The Pareto charts of each algorithm showed that the algorithms with differential evolution would improve their global search capability and convergence, and NSGA-II-DE performed the best. Secondly, a triple-column distillation sequence in the publication was optimized by using NSGA-II-DE. By optimizing the feed stage, the number of trays, and operative pressure, the CO_2_ emission and total annual cost were reduced by 64.15% and 82.23%, respectively. This study demonstrated the ability of NSGA-II-DE to obtain the optimal synthesis of the distillation sequence and explores an overall solution of distillation separation design.

## Figures and Tables

**Table 1:** Properties of the fourteen-component mixture.

Component ID	Component	Molar Feeds (kmol/h)	*K*-Values	Boiling Points (°C)
A	methane	230	256.99	−161.49
B	ethane	100	93.25	−88.60
C	propane	40	45.63	−42.04
D	*n*-butane	50	22.37	−0.50
E	*n*-pentane	130	11.34	36.07
F	*n*-hexane	100	5.86	68.73
G	*n*-heptane	110	3.08	98.43
H	*n*-octane	180	1.61	125.68
I	*n*-nonane	120	0.86	150.82
J	*n*-decane	30	0.46	174.16
K	*n*-undecane	150	0.26	195.93
L	*n*-dodecane	190	0.14	216.32
M	*n*-tridecane	90	0.07	235.47
N	*n*-tetradecane	140	0.04	253.58

**Table 2:** The parameters of six evolution algorithms.

Evolution Algorithm	Parameters
NSGA-II	*dim* = 2; *size* = 100; *gen* = 100; *cf* = 1; *mf* = 1/dim
NSGA-III	*dim* = 2; *size* = 100; *gen* = 100; *cf* = 1; *mf* = 1/*dim*
MOEA/D	*dim* = 2; *size* = 100; *gen* = 100; *cf* = 1; *mf* = 1/*dim*; *ps* = 0.9; *sn* = *size*/10
NSGA-II-DE	*dim* = 2; *size* = 100; *gen* = 100; *f* = 0.5; *Cr* = 0.5 *mf* = 1/*dim*; *ps* = 0.9
NSGA-III-DE	*dim* = 2; *size* = 100; *gen* = 100; *f* = 0.5; *Cr* = 0.5 *mf* = 1/*dim*; *ps* = 0.9
MOEA/D-DE	*dim* = 2; *size* = 100; *gen* = 100; *f* = 0.5; *Cr* = 0.5 *mf* = 1/*dim*; *sn* = *size*/10

**Table 3:** NRTL parameters for binary systems.

Component *i*	Component *j*	*A_ij_*	*A_ji_*	*B_ij_*	*B_ji_*
Acetone	Isopropanol	−2.4106	2.4494	822.4892	−583.3452
Acetone	Water	6.3981	0.0544	−1808.9910	419.9716
Isopropanol	Water	−1.3115	6.8284	426.3978	−1483.4573
Acetone	MIBK	−5.4452	5.3013	1833.5227	−1735.9082
Isopropanol	MIBK	0.0000	0.0000	160.6435	28.1164
Water	MIBK	9.1629	−3.2305	−1248.7440	1208.8770
Water	MIBC	10.2983	−3.2359	−1367.8159	998.0640
Water	DIBK	11.6082	−0.3283	−969.9380	730.5226
MIBK	MIBC	0.3818	−0.1565	0.0000	0.0000
Acetone	MIBC	0	0	222.1975	7.9431
Acetone	DIBK	0	0	335.0488	−164.9281
Isopropanol	MIBC	0	0	159.3051	−122.9533
Isopropanol	DIBK	0	0	263.2273	125.6002
MIBK	DIBK	0	0	123.9190	−77.4980
MIBC	DIBK	0	0	89.2102	172.8563

**Table 4:** Range of variation in population individual.

Decision Variable	Variable Category	Change Range
T1 total number of trays	integer	[30,60]
T1 ratio of the feed stage to the total number of trays	real number	[0.1,0.95]
T1 operative pressure	integer	[40,100]
T2 total number of trays	integer	[30,60]
T2 ratio of the feed stage to the total number of trays	real number	[0.1,0.95]
T2 operative pressure	integer	[40,85]
T3 total number of trays	integer	[30,60]
T3 ratio of the feed stage to the total number of trays	real number	[0.1,0.95]
T3 operative pressure	integer	[60,100]

**Table 5:** Comparison between initial results and NSGA-II-DE optimization results.

Operation Parameters	Base Case [29]	After Optimization (Min Euclidean Distance)
T1 total number of trays	35	56
T1 feed stage	30	47
T1 operative pressure (kPa)	101.32	100
T2 total number of trays	36	43
T2 feed stage	31	37
T2 operative pressure (kPa)	70.93	58
T3 total number of trays	32	58
T3 feed stage	10	23
T3 operative pressure (kPa)	70.93	100
TAC (million$)	2.912	1.044
CO_2_ Emission (kt/year)	6.083	1.083

## Data Availability

The data presented in this study are available on request from the corresponding author.

## Sample Availability

Samples of the compounds are not available from the authors.
